# Genetic–Epigenetic Interactions in Uterine Leiomyomas: *MED12* Mutations as Predictors of Aberrant DNA Methylation

**DOI:** 10.3390/genes17080877

**Published:** 2026-07-28

**Authors:** Tayyaba Kazmi, Ruqaya Nangrejo, Eraj Abbas, Nazish Waris, Iftikhar Ahmed Siddiqui, Rehana Rehman, Paul Laurance-Young

**Affiliations:** 1 Faculty of Basic Medical Sciences, Baqai Medical University, Karachi 75340, Pakistan; ruqayakhan@baqai.edu.pk (R.N.); nazishwaris@baqai.edu.pk (N.W.); dr.iftikhar@baqai.edu.pk (I.A.S.); 2 Faculty of Health Sciences, Aga Khan University and Hospital, Karachi 74800, Pakistan; rehana.rehman@aku.edu; 3 School of Biomedical Sciences, University of Plymouth, Portland Square, Plymouth PL4 8AA, UK; paul.laurance-young@plymouth.ac.uk

**Keywords:** uterine fibroids, *MED12* gene, polymorphism, DNA methylation

## Abstract

**Objective:** The study was conducted to investigate the frequency of *MED12* gene (rs5030619) mutations and to evaluate DNA methylation patterns in uterine leiomyomas among women at a tertiary care hospital in Karachi, Pakistan. **Methods:** In this cross-sectional study, 200 women with uterine fibroids and 50 controls were recruited from tertiary care hospitals after ethical approval. Baseline data and history were collected. Serum levels of estradiol, progesterone, and FSH were measured. Genomic DNA was extracted from fibroid and myometrial tissues and analyzed using allele-specific PCR for *MED12* genotyping. DNA methylation profiling was conducted to evaluate epigenetic modifications. Statistical analyses were performed using the Statistical Package for social sciences (SPSS) version 27.0. **Results:** The majority of cases were aged 31–50 years, with 41.5% overweight and 24.5% obese. An increased WHR was observed in 65% of cases, compared to only 36% of controls. Serum estradiol, progesterone, and FSH levels were significantly higher in cases across all menstrual phases (*p* < 0.01). The *MED12* A/C genotype was significantly associated with fibroid risk (OR = 11.72, 95% CI: 5.59–24.57, *p* < 0.001), and the C/C genotype conferred the highest risk (OR = 18.17, 95% CI: 4.00–82.54, *p* = 0.001). C allele was associated with increased disease susceptibility (OR = 4.65, 95% CI: 2.69–8.03, *p* < 0.001). Hyper-methylation was the most prevalent finding, observed in 125 cases of leiomyomas (69.4%). Multivariable logistic regression identified age (OR = 1.04, *p* = 0.012), BMI (OR = 1.09, *p* = 0.004), multiple fibroids (OR = 2.31, *p* = 0.005), intramural fibroid location (OR = 1.88, *p* = 0.041), and positive family history (OR = 2.74, *p* = 0.003) as significant independent predictors. **Conclusions:** Uterine leiomyomas may be influenced by a combination of hormonal, metabolic, genetic, and epigenetic factors. Overall findings suggest that *MED12* C allele and A/C genotype variation was significantly associated with increased DNA methylation in uterine leiomyomas.

## 1. Introduction

Uterine leiomyomas, or uterine fibroids, are non-malignant tumors arising from the smooth muscle layer of the uterus and represent the most common neoplastic disorder among women of reproductive age [1]. It is characterized by excessive extracellular matrix (ECM) deposition, increased smooth muscle cell proliferation, and a sustained inflammatory environment within the myometrium [2]. Hormonal factors, particularly estrogen and progesterone, play a central role in fibroid development and growth [3]. Benign leiomyomas are a significant cause of gynecologic morbidity and can cause abnormal uterine bleeding (menorrhagia), pelvic pain, fertility complications and/or recurrent pregnancy loss, and adverse obstetric outcomes [4]. Although leiomyomas are very common and clinically important, the molecular basis behind their development and progression is not fully understood.

There is growing evidence that the origin of uterine fibroids is a complex interaction of genetic, hormonal, environmental, and epigenetic factors [3]. One of the genetic changes found is a mutation in the mediator complex subunit 12 (*MED12*) gene, which is the most prevalent somatic mutation in uterine leiomyomas, found in around 60–70% of all cases. *MED12* encodes an essential regulatory component of the mediator complex and functions as part of the kinase module of the mediator complex, together with MED13, cyclin C, and cyclin-dependent kinases CDK8 or CDK19 [5,6]. The disruption of *MED12* activity has been associated with various benign and malignant human tumors [7]. *MED12* is one of the most extensively studied genes in uterine leiomyoma pathogenesis. Recurrent somatic mutations in exon 2 of MED12 are detected in approximately 60–70% of leiomyomas and are considered major tumor-specific driver mutations [8]. These alterations are confined to leiomyoma tissue and are frequently linked to smaller tumor size, subserosal location, and the presence of multiple fibroids within the same uterus [9,10]. Although somatic *MED12* exon 2 mutations are well-established drivers of uterine leiomyoma pathogenesis, the role of inherited *MED12* polymorphisms in disease susceptibility has not been fully elucidated.

Beyond the genetic abnormalities, epigenetic changes are also now recognized as playing a role in the pathogenesis of leiomyoma. Among the most well-studied epigenetic modifications, DNA methylation is an important epigenetic regulator of gene expression, chromatin structure, cellular differentiation, and tissue remodeling [11]. In uterine leiomyomas, abnormal methylation patterns were found and linked to tumor initiation and progression by silencing tumor suppressor genes and/or by disrupting pathways that regulate cell growth and extracellular matrix accumulation [12].

The association between *MED12* mutations and DNA methylation is still under investigation. Recent findings have shown a possible difference in epigenetic profiles between *MED12*-mutated and non-mutated leiomyomas [13], leading to the hypothesis that genetic and epigenetic mechanisms may work together in the genesis of these neoplasms. In addition, epigenetic changes have been shown to be associated with age and obesity in patients, and could potentially affect the DNA methylation of patients with leiomyoma [14].

The present study was conducted to investigate the frequency of *MED12* gene mutations and evaluate DNA methylation patterns in uterine leiomyomas among women undergoing surgical treatment at a tertiary care hospital in Karachi, Pakistan. Furthermore, the study aimed to examine the association between *MED12* mutation status, DNA methylation levels, and selected clinicopathological characteristics to provide insights into the genetic and epigenetic mechanisms underlying uterine leiomyoma pathogenesis.

## 2. Materials and Methods

This study was conducted as a cross-sectional study from April 2024 to December 2025, following approval from the Institutional Review & Ethics Board (IREB) with approval number BMU-IREB/06-2024/009. Informed written consent was obtained from each participant of this study prior to their sample collection. Participants were recruited from Fatima Hospital, Baqai Medical University, and Aga Khan University Hospital as tertiary care centers providing specialized gynecological services. The sample size was calculated using OpenEpi version 3.0.1, and the calculated sample size was *n* = 178 participants. To improve statistical robustness, a total of *n* = 250 female patients aged 22 years and above were included using a non-probability convenience sampling method. This study included 200 women as cases diagnosed with uterine fibroids and 50 healthy women as controls. It included women aged 22 years and above with a confirmed diagnosis of uterine fibroids. The diagnosis was based on clinical assessment and radiological confirmation using pelvic ultrasonography (transabdominal and/or transvaginal). In cases undergoing surgery, histopathological examination further confirmed the presence of benign smooth muscle tumors consistent with leiomyomas. Healthy controls were included with no history of uterine fibroids. The unequal case-to-control ratio (4:1) was primarily due to the limited availability of control subjects without fibroids. The participants with the presence of other gynecological conditions such as polycystic ovarian syndrome, endometriosis, ovarian cysts, or tumors, and current use of hormonal therapy were excluded. The data were collected using a structured questionnaire, and a detailed medical history and clinical assessment were documented.

**Figure 1:**
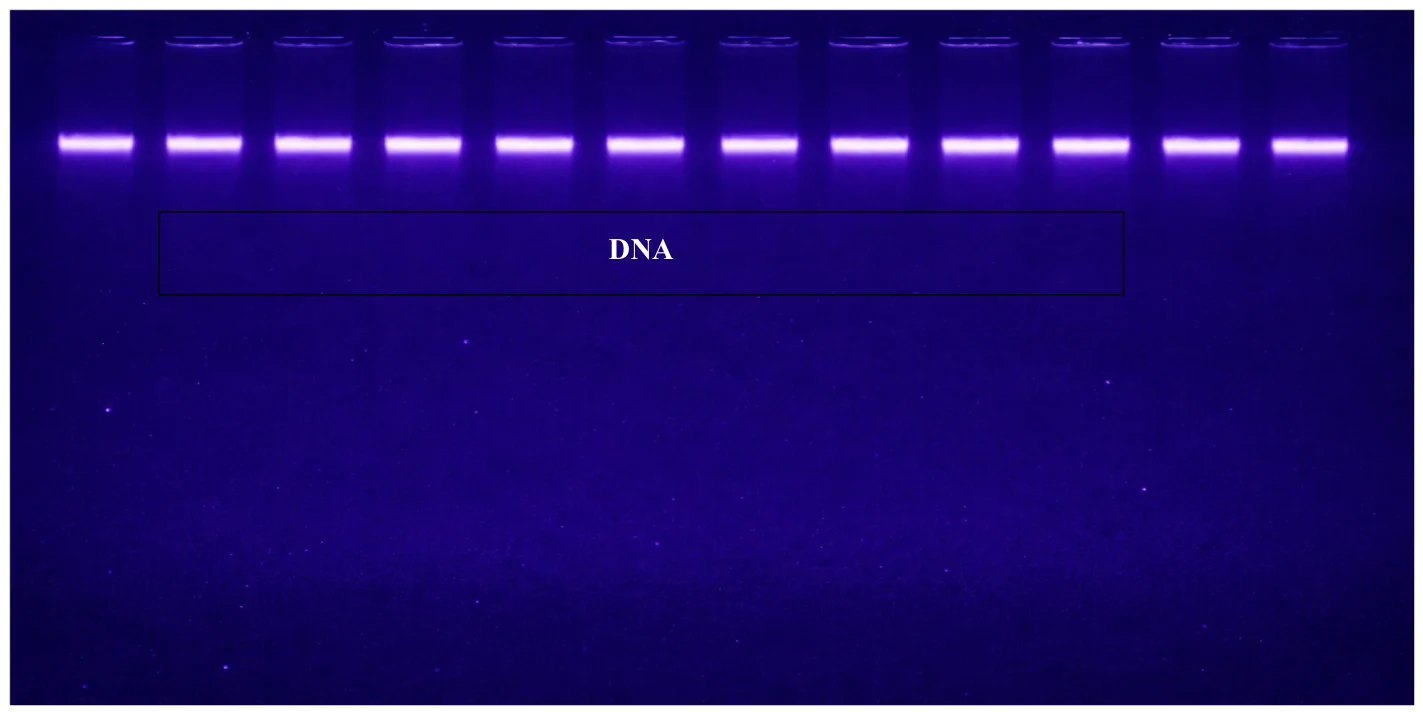
Whole Genomic DNA extraction.

Biophysical measurements included weight, height, body mass index (BMI), waist and hip circumference, waist-to-hip ratio (WHR), and fibroid characteristics such as size and anatomical location. A venous blood sample (5 mL) was collected under aseptic conditions, with 3 mL stored for molecular analysis and 2 mL used for biochemical assays. Serum levels of follicle-stimulating hormone (FSH), estrogen, and progesterone were measured using an ELISA kit (Invitrogen™, ThermoFisher Scientific, Waltham, MA, USA). DNA was extracted by the standard salting-out method from peripheral blood samples. The integrity of genomic DNA was qualitatively assessed by gel electrophoresis as illustrated in Figure 1. Genotyping of the *MED12* single-nucleotide polymorphism rs5030619 (A>C) was performed from peripheral blood-derived gDNA using the allele-specific polymerase chain reaction (AS-PCR) technique, also referred to as amplification refractory mutation system (ARMS) PCR. This technique is based on the principle that a 3′ terminal mismatch between the primer and the template significantly reduces or terminates PCR amplification efficiency under optimized cycling conditions [15], thereby permitting discrimination between the wild-type (A) and mutant (C) alleles in a single reaction. A total of 250 participants were recruited for the study, but DNA methylation analysis was performed on a subset of 180 participants with sufficient high quality DNA. Therefore, analyses involving methylation measures are based on this subset. DNA methylation status of the *MED12* gene was carried out using Combined Bisulfite Restriction Analysis (COBRA), a semi-quantitative technique to determine the proportion of methylated cytosine at specific CpG sites. Bisulfite-treated DNA was amplified using primers specifically designed to hybridize with bisulfite-converted sequences, in which non-CpG cytosine is replaced by thymine to ensure sequence complementarity. Amplification was carried out under standard PCR conditions using one unit (1 U per reaction) of Biotaq HS DNA polymerase (Bioline Ltd., London, UK) in a 25 µL reaction volume containing the corresponding primer pairs. After PCR amplification, 10 µL of PCR product was digested with either HpyCH4IV (New England Biolabs, Ipswich, MA, USA) or TaqI (Takara Bio Inc., Tokyo, Japan), enzyme selection determined by the recognition site present at each target CpG locus. Restriction digestion was performed at enzyme-specific optimal temperature (HpyCH4IV: 37 °C; TaqI: 65 °C) for 2–4 h using the manufacturer’s recommended buffer. Band intensities were quantified by densitometry using ImageJ (v1.53; NIH, Bethesda, MD, USA). Methylation levels were calculated by densitometric analysis using the formula:

Methylation (%) = [(Intensity of digested band)/(Intensity of digested band + Intensity of undigested band)] × 100

Based on methylation score distributions observed in the dataset, samples were categorized as follows:

Hypermethylated (methylation score ≥ 0.8).

1. Hypomethylated (score 0.10–0.79)
2. Mildly methylated (score 0.01–0.09)
3. Unmethylated (score ≈ 0.00)

The data was analyzed using statistical package for the social sciences (SPSS) statistics version 27.0, and R software, version 2.14.0 (www.r-project.org, accessed on 14 September 2025). Continuous variables were expressed as mean ± standard deviation. The normality of the continuous variables was checked using the Shapiro–Wilk test. As the study variables satisfied the assumption of normality, parametric statistical tests were used for analysis. Differences in continuous variables between cases and controls were assessed using an independent *t*-test. Categorical variables were compared using the Chi-square test. Variables with significant associations in univariate analyses were further evaluated using multivariate logistic regression to determine independent predictors of MED12 mutation status. Genotype and allele frequencies for the *MED12* gene were analyzed using the SNPStats web-based tool, and odds ratios with 95% confidence intervals were calculated under codominant, dominant, recessive, and overdominant genetic models.

## 3. Results

Table 1 presents the anthropometric and clinical characteristics of study participants, and it was seen that the maximum number of cases was observed in the age group of 41–50 years (37.5%), followed by 31–40 years (29.5%) as compared to controls. In the present study, a higher proportion of leiomyoma cases belonged to the overweight (41.5%) and obese (24.5%) participants as compared to controls (18% and 10%, respectively). Normal BMI was more commonly observed among controls (64%) than cases (28.5%). An increased WHR was observed in 65% of cases, compared to only 36% of controls. Conversely, a normal WHR was more frequent among controls (64%) than cases (35%).

Table 2 shows the distribution of uterine leiomyomas according to tumor type, number of fibroids, and duration of disease among the study participants. Among the 200 women diagnosed with uterine leiomyoma, the majority had intramural leiomyomas (61.5%). Regarding the number of leiomyomas, multiple leiomyomas were observed in 62.5% of participants. In terms of disease duration, most participants had been diagnosed with leiomyoma for 1–3 years (42.5%).

Table 3 shows the comparison of FSH, Estradiol, and Progesterone levels among premenopausal, postmenopausal, and elderly female cases and control. Significantly higher levels of these hormonal levels was found among cases in all menstrual phases (*p* < 0.01).

**Figure 2:**
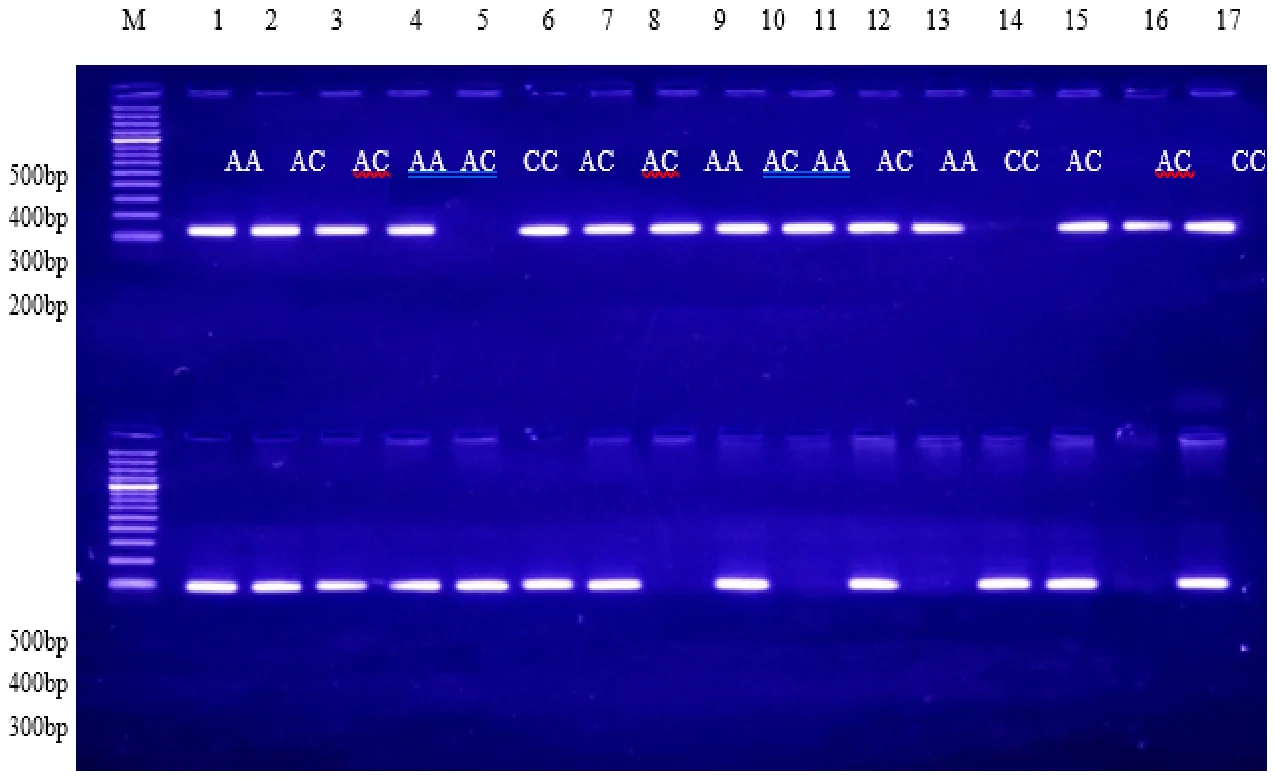
Genotyping of *MED12* (rs5030619) variant by AS-PCR.

Genotyping of the MED12 (rs5030619) variant by PCR is shown in Figure 2. Lanes 1, 4, 9, 11, and 13 depicted the genotype of mutant homozygous (A/A). Lanes 6, 14, and 17 represented the genotype of wild-type homozygous (C/C), while lanes 2, 3, 5, 7, 8, 10, 12, 15, and 16 indicated the genotypes of heterozygosity (A/C).

**Figure 3:**
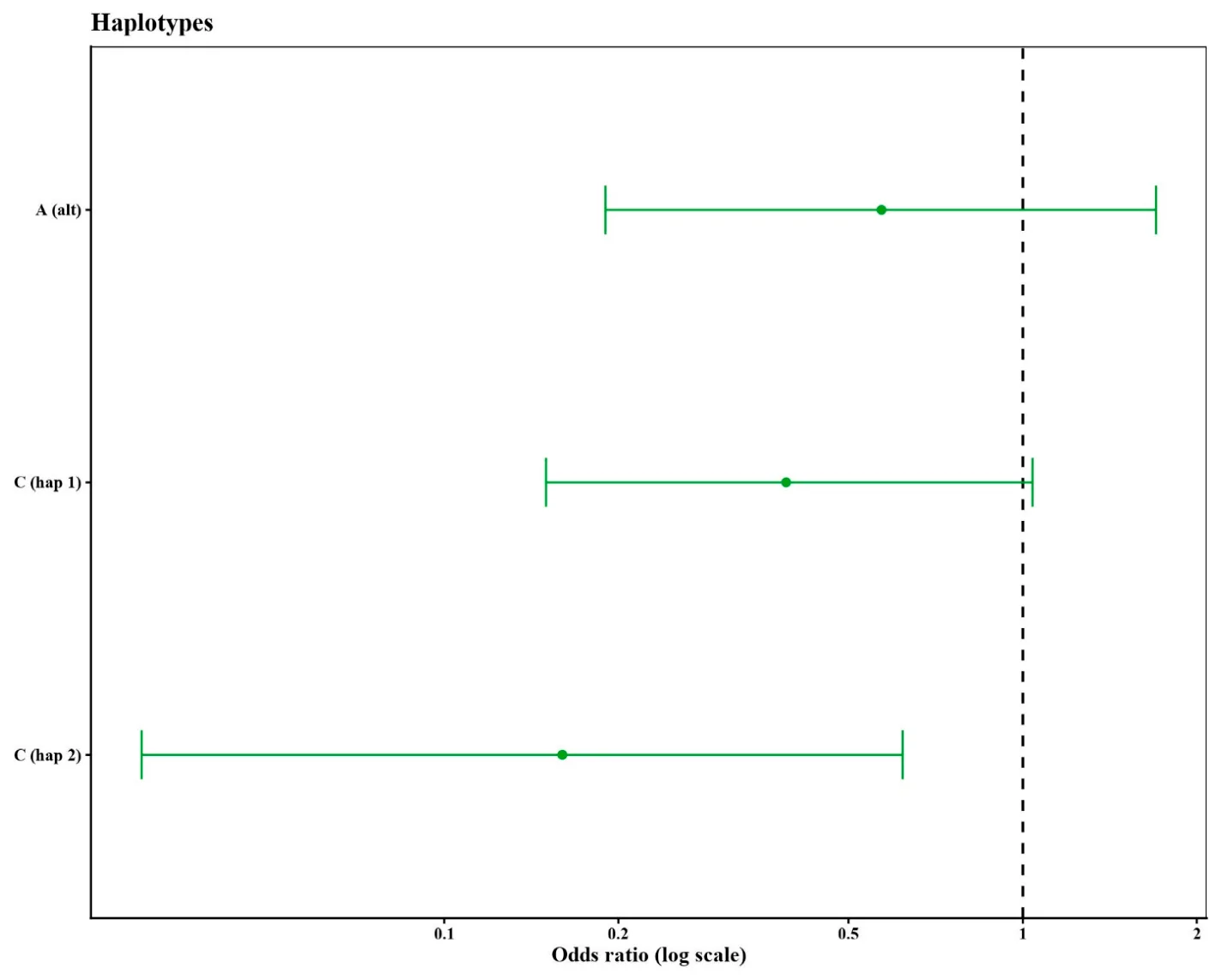
Haplotype frequencies of the MED12 A/C variants.

Figure 3 depicts the haplotype frequencies of the MED12 A/C variants in cases and controls and their association with disease risk. Haplotype frequencies are shown for both groups, with odds ratios (ORs) and 95% confidence intervals (CIs) calculated to estimate the strength of association. The most frequent haplotype in cases was used as the reference category (OR = 1.00). Statistical significance was assessed, and corresponding *p*-Values are reported. Among the MED12 haplotypes, the A haplotype showed higher frequency in cases compared with controls, while certain C haplotypes were more frequent in controls. One C haplotype demonstrated a statistically significant protective association against disease (OR = 0.16, 95% CI: 0.03–0.62, *p* = 0.001), whereas another C haplotype showed a borderline association (OR = 0.39, 95% CI: 0.15–1.04, *p* = 0.05). No significant association was observed for the second A haplotype (*p* = 0.30).

**Figure 4:**
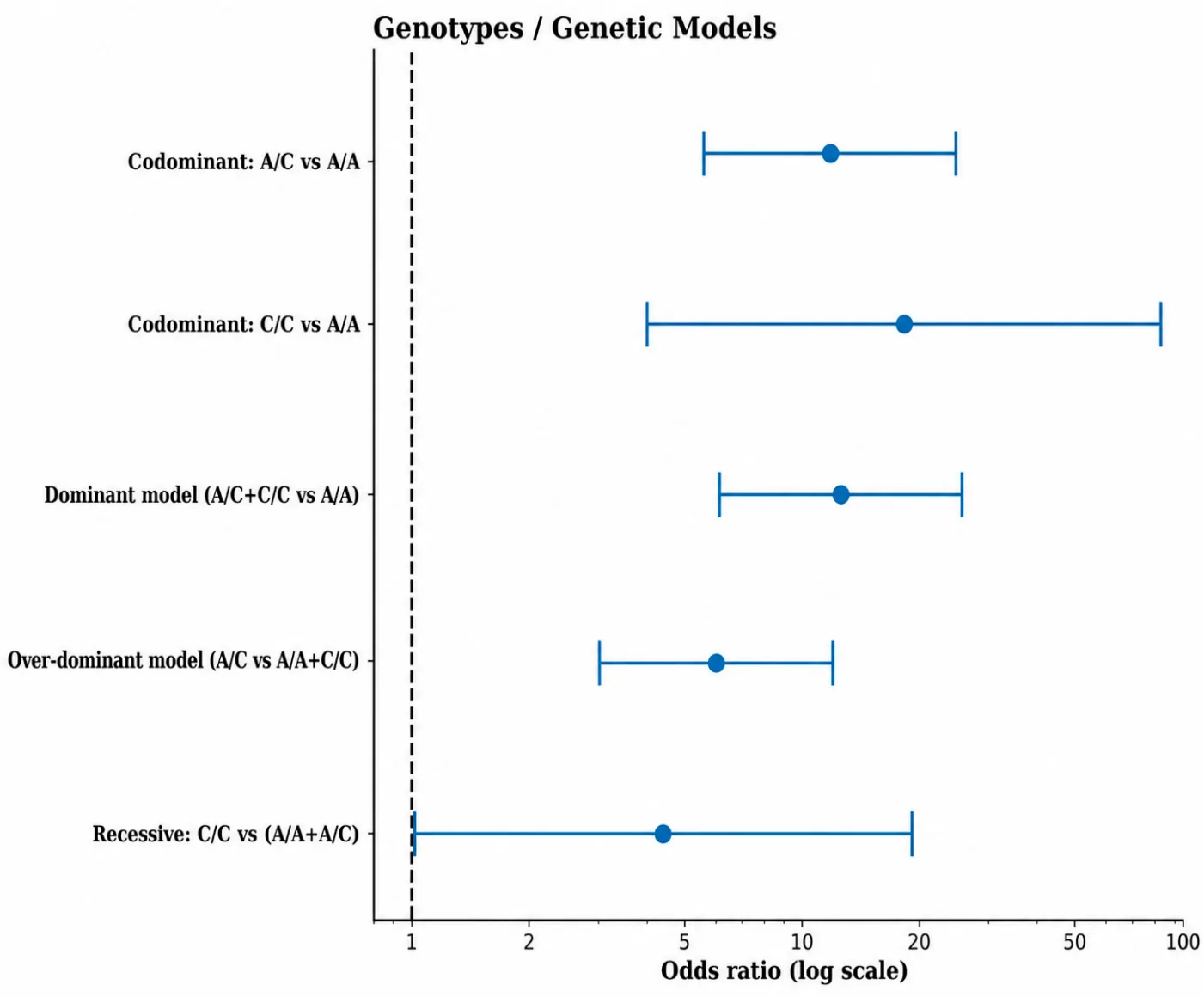
Distribution of MED12 A/C Genotypes.

Figure 4 presents the distribution of MED12 A/C genotypes under different genetic models. Codominant model: A/C vs. A/A, OR = 11.72 (95% CI 5.59–24.57); C/C vs. A/A, OR = 18.17 (95% CI 4.00–82.54). Dominant model (A/C+C/C vs. A/A): OR = 12.53 (95% CI 6.14–25.56). Overdominant model (A/C vs. A/A+C/C): OR = 6.00 (95% CI 3.02–11.93). Recessive model (C/C vs. A/A+A/C): OR = 4.40 (95% CI 1.02–19.06). A/A served as the reference genotype for the codominant models. The dashed vertical line at OR = 1 indicates no association; point estimates are plotted on a log scale with horizontal bars representing 95% CIs.

Table 4 presents the distribution of MED12 A/C genotypes and alleles among cases (*n* = 200) and controls (*n* = 50). Among the genotypes, the A/C heterozygous genotype was the most common in cases, occurring in 140 (70.0%) participants, compared with only 14 (28.0%) controls. In contrast, the A/A genotype was observed more frequently in controls, with 34 (68.0%) individuals, than in cases, where it was identified in 29 (14.5%) participants. The C/C genotype was relatively uncommon but was more frequently detected in cases (31; 15.5%) than in controls (2; 4.0%). Overall, the genotype distribution differed significantly between cases and controls (χ^2^ = 60.90, df = 2, *p* < 0.0001).

For the allelic analysis, 400 alleles from cases and 100 alleles from controls were evaluated. The C allele was more common in cases (202; 50.5%) than in controls (18; 18.0%), whereas the A allele was observed more frequently in controls (82; 82.0%) than in cases (198; 49.5%). Individuals carrying the C allele had a significantly higher likelihood of being in the case group, with an odds ratio (OR) of 4.65 (95% CI: 2.69–8.03). These findings indicate a significant association between the MED12 C allele and disease susceptibility (*p* < 0.0001), suggesting that this allele may contribute to an increased risk of disease.

Table 5 shows the frequency distribution of DNA methylation patterns, and it is seen that hyper-methylation was the most prevalent finding, observed in 125 cases (69.4%). This was followed by hypo-methylation, identified in 38 cases (21.1%), and mild methylation, present in 14 cases (7.8%). No methylation was observed in only three cases (1.7%), indicating that the vast majority of samples exhibited some degree of DNA methylation.

Table 6 shows the distribution of DNA methylation scores across different methylation ranks. Hypermethylated regions constituted the majority and demonstrated high methylation scores with minimal variability, indicating consistent epigenetic silencing. Hypomethylated regions showed moderate methylation scores with greater variability. Mildly methylated and no methylated regions accounted for a small proportion of the total regions and exhibited very low methylation scores. This distribution highlights the predominance of hyper-methylation events within the studied fibroid samples.

Table 7 presents the multivariate logistic regression analysis evaluating factors independently associated with the (rs5030619) mutation in the *MED12* gene. Age was significantly associated with increased odds of the outcome, with each additional year increasing the likelihood by 4% (OR = 1.04, 95% CI: 1.01–1.07, *p* = 0.012). Similarly, body mass index (BMI) was a significant predictor, as each one-unit increase in BMI was associated with a 9% increase in the odds of the outcome (OR = 1.09, 95% CI: 1.03–1.16, *p* = 0.004). Women with multiple fibroids had significantly higher odds of the outcome compared to those with a single fibroid, demonstrating more than a two-fold increase in risk (OR = 2.31, 95% CI: 1.28–4.17, *p* = 0.005). The presence of intramural fibroids was also independently associated with the outcome, increasing the odds by approximately 88% compared to other fibroid locations (OR = 1.88, 95% CI: 1.02–3.44, *p* = 0.041). Positive family history presented as a strong predictor, with affected individuals exhibiting nearly three times higher odds of the outcome than those without a family history (OR = 2.74, 95% CI: 1.36–5.52, *p* = 0.003).

Table 8 shows the linear regression analysis to evaluate the factors associated with DNA methylation levels in cases, and it was observed that (rs5030619) mutations in *MED12* were found to be a significant predictor of increased DNA methylation levels, with mutated fibroids exhibiting an average increase of 4.6 units in methylation (β = +4.6, SE = 1.2, *p* < 0.001). Age was also significantly associated with DNA methylation levels. For each additional year of age, DNA methylation increased by 0.08 units (β = +0.08, SE = 0.03, *p* = 0.011), indicating a positive relationship between advancing age and methylation status in fibroid tissue. BMI also demonstrated a significant positive association with DNA methylation levels. Each one-unit increase in BMI was associated with a 0.15-unit increase in DNA methylation (β = +0.15, SE = 0.05, *p* = 0.006).

## 4. Discussion

The present study investigated *MED12* gene (rs5030619) mutations and DNA methylation patterns in patients with uterine leiomyomas. Our findings demonstrated that *MED12* mutations were highly prevalent among leiomyoma tissues, with the majority of mutations occurring in the heterozygous A/C genotype. Furthermore, epigenetic analysis revealed a predominance of DNA hyper-methylation in leiomyoma samples, suggesting that both genetic and epigenetic mechanisms contribute to the pathogenesis of uterine fibroids.

*MED12* is one of the most frequently mutated genes in uterine leiomyomas and plays a critical role in transcriptional regulation through the mediator complex [16]. The present study demonstrated a significant association between the *MED12* rs5030619 polymorphism and susceptibility to uterine leiomyoma in our study population. Alternatively, a previous study has identified that somatic *MED12* mutations are among the most common molecular alterations in uterine leiomyomas, being identified in approximately 60–80% of tumor tissues [17]. The haplotype analysis of *MED12* A/C variants in this study reveals distinct genetic patterns between cases and controls, suggesting differential allelic contributions to uterine leiomyoma susceptibility. The most frequent C haplotype predominated among cases, consistent with previous observations that *MED12* A haplotype alterations are highly prevalent in leiomyomas and represent primary genetic events in tumorigenesis [18].

Allelic analysis of the *MED12* gene further reinforced these findings, with the C allele being significantly more frequent among women with uterine leiomyoma than controls. This finding suggests that the C allele may confer increased genetic susceptibility to fibroid development in the studied population. The previous studies indicated that *MED12* mutations are highly recurrent in uterine fibroids and may act as primary drivers of tumor initiation and growth [19]. These findings are also consistent with emerging evidence that *MED12* mutations contribute to transcriptional repression and modulate cellular pathways involved in smooth muscle proliferation and extracellular matrix accumulation in fibroids [20,21,22].

In addition to genetic alterations, our study demonstrated significant DNA hyper-methylation in leiomyoma tissues. DNA methylation is a major epigenetic mechanism regulating gene expression, and aberrant methylation can lead to the silencing of tumor suppressor genes and dysregulation of cellular pathways involved in growth and differentiation [23]. The observed DNA hyper-methylation in the present study may contribute to leiomyoma development by suppressing the expression of genes involved in cell-cycle regulation, apoptosis, hormone responsiveness, and extracellular matrix remodeling. Similar epigenetic alterations have been reported in previous studies, supporting the hypothesis that aberrant DNA methylation is a key molecular mechanism underlying uterine leiomyoma pathogenesis [11,24,25]. The coexistence of *MED12* mutations and DNA hyper-methylation in our study suggests a possible interaction between genetic and epigenetic events during tumor development. *MED12* mutations may create a cellular environment that favors epigenetic alterations, while abnormal methylation patterns may further enhance the proliferative advantage of mutated cells. This synergistic effect could explain the initiation and progression of uterine leiomyomas and underscores the complexity of their molecular pathogenesis.

The present study demonstrated that age, BMI, multiple fibroids, intramural fibroid location, and a positive family history were significant predictors of uterine leiomyomas. Furthermore, age, BMI, and *MED12* mutation status were independently associated with increased DNA methylation levels, suggesting an interplay between clinical, genetic, and epigenetic mechanisms in fibroid pathogenesis. Increasing age was associated with both a higher likelihood of leiomyoma occurrence and elevated DNA methylation levels. Increasing age was associated with both a higher likelihood of leiomyoma occurrence and elevated DNA methylation levels. These findings are consistent with previous studies indicating that prolonged exposure to ovarian hormones and the cumulative accumulation of genetic and epigenetic alterations contribute to fibroid development [26,27]. Age-related changes in DNA methylation, commonly referred to as epigenetic drift, have been implicated in altered gene expression and cellular proliferation, providing a potential mechanism linking aging to leiomyoma growth [28].

Similarly, higher BMI was significantly associated with both leiomyoma risk and increased DNA methylation levels. Obesity is a well-established risk factor for uterine fibroids and may promote tumor growth through increased peripheral estrogen production, insulin resistance, chronic inflammation, and altered adipokine secretion [29]. These metabolic disturbances have also been shown to influence epigenetic regulation, leading to aberrant DNA methylation patterns that may contribute to dysregulated cellular proliferation and extracellular matrix production within fibroid tissues. The concurrent association of BMI with both disease risk and methylation status highlights a potential mechanistic link between metabolic factors and epigenetic alterations in leiomyomas.

A positive family history emerged as one of the strongest predictors of leiomyoma susceptibility, supporting the contribution of inherited genetic factors to disease development. Familial aggregation of uterine fibroids has been reported in previous, suggesting that genetic predisposition may influence individual susceptibility to tumor formation [30]. Likewise, women with multiple fibroids exhibited significantly greater odds of disease, which may reflect an underlying genetic tendency toward abnormal myometrial cell proliferation and clonal expansion. Intramural fibroids were also independently associated with the outcome, possibly owing to their location within the myometrium, where hormonal responsiveness and growth potential may be enhanced.

Importantly, *MED12* mutation status was identified as the strongest predictor of increased DNA methylation levels. Fibroids harboring *MED12* mutations demonstrated significantly higher methylation levels than non-mutated tumors, suggesting that *MED12* alterations may influence epigenetic regulation. As a key component of the mediator transcription complex, *MED12* plays a critical role in gene expression regulation, and its disruption may induce epigenetic modifications that facilitate leiomyoma growth and maintenance. The strong association between *MED12* mutations and DNA methylation observed in the present study supports the hypothesis that genetic and epigenetic mechanisms act synergistically in the pathogenesis of uterine leiomyomas.

Taken together, these findings indicate that age and obesity may contribute to both the development of uterine leiomyomas and the epigenetic alterations observed within fibroid tissues, while *MED12* mutations appear to represent a critical molecular driver of aberrant DNA methylation. The interaction between these clinical, genetic, and epigenetic factors may play a central role in leiomyoma initiation and progression.

Collectively, these findings suggest that both genetic alterations and host-related factors contribute to the epigenetic landscape of uterine leiomyomas. The significant association between *MED12* mutations and elevated DNA methylation levels supports the hypothesis that genetic and epigenetic mechanisms interact in the pathogenesis of fibroids. Further studies incorporating genome-wide methylation profiling and functional analyses are warranted to elucidate the specific pathways through which these factors may influence leiomyoma development and progression.

The findings of this study have important clinical implications. Identification of *MED12* mutations and specific methylation signatures may serve as valuable molecular biomarkers for the diagnosis and classification of uterine leiomyomas. Moreover, epigenetic alterations are potentially reversible, raising the possibility of targeted therapeutic interventions using epigenetic-modifying agents in the future.

## 5. Conclusions

In conclusion, our study demonstrated a high prevalence of the rs5030619 mutation in the *MED12* gene, particularly the A/C genotype, along with widespread DNA hyper-methylation in uterine leiomyoma patients. These findings support that both genetic and epigenetic mechanisms may be involved in leiomyoma development and provide further insight into the molecular basis of this common gynecological tumor.

## Figures and Tables

**Table 1:** Anthropometric characteristics of study participants.

Variable	Category	Cases (*n* = 200)	Controls (*n* = 50)
Age (years)	22–30	19 (9.5%)	10 (20%)
	31–40	59 (29.5%)	13 (26%)
	41–50	75 (37.5%)	7 (14%)
	51–60	25 (12.5%)	9 (18%)
	61–70	15 (7.5%)	5 (10%)
	71–80	7 (3.5%)	6 (12%)
BMI (kg/m^2^)	<18.5 (Underweight)	11 (5.5%)	4 (8%)
	18.5–24.9 (Normal)	57 (28.5%)	32 (64%)
	25–29.9 (Overweight)	83 (41.5%)	9 (18%)
	≥30 (Obese)	49 (24.5%)	5 (10%)
Waist–Hip Ratio (WHR)	Normal	70 (35%)	32 (64%)
	Increased	130 (65%)	18 (36%)

Data presented as *n* (%).

**Table 2:** Clinical characteristics of uterine leiomyomas (*n* = 200).

Variable	Category	Frequency (*n*)	Percentage (%)
Type of Leiomyoma	Intramural	123	61.5
	Subserosal	47	23.5
	Submucosal	30	15.0
Number of Leiomyomas	Single	75	37.5
	Multiple	125	62.5
Duration of Leiomyoma (Years)	<1	27	13.5
	1–3	85	42.5
	4–6	60	30.0
	>6	28	14.0

Data presented as *n* (%).

**Table 3:** Comparison of hormonal levels between cases and controls.

Variable	Category	Cases (*n* = 200)	Controls (*n* = 50)	*p*-Value
Follicle-Stimulating Hormone (FSH) pg/mL	Premenopausal Females (41–50 Years)	14 ± 3	11.5 ± 3	0.004
	Postmenopausal Females (>50 Years)	55 ± 11	23 ± 7	<0.01
	Elderly Females (65–80 Years)	71 ± 12	37 ± 9	<0.01
Estradiol (IU/L)	Premenopausal Females (41–50 Years)	159 ± 17	140 ± 15	0.001
	Postmenopausal Females (>50 Years)	19 ± 6	10 ± 3	<0.01
	Elderly Females (65–80 Years)	5 ± 1	2 ± 1.15	<0.01
Progesterone (ng/mL)	Premenopausal Females (41–50 Years)	2 ± 0.5	1.4 ± 0.4	0.003
	Postmenopausal Females (>50 Years)	0.2 ± 0.1	0.01 ± 0.001	<0.01
	Elderly Females (65–80 Years)	0.1 ± 0.1	0.01 ± 0.001	<0.01

**Table 4:** Distribution of MED12 A/C between cases and controls.

Genotypes *n* = 250	Cases *n* = 200	Controls *n* = 50	Chi Square *p*-Value
A/A	29 (14.5%)	34 (68.0%)	χ^2^ = 60.90, df = 2 *p* < 0.0001
A/C	140 (70.0%)	14 (28.0%)	
C/C	31 (15.5%)	2 (4.0%)	
**Alleles *n* = 500**	***n*** ** = 400**	***n*** ** = 100**	**OR (95% CI)**
A	198 (49.5%)	82 (82.0%)	1.00
C	202 (50.5%)	18 (18.0%)	4.65 (2.69–8.03)

**Table 5:** Association of DNA methylation of *MED12* gene in uterine leiomyomas.

Frequency (%)	No Methylation	Hyper-Methylation	Mild Methylation	Hypo-Methylation
*n*	3	125	14	38
%	1.7	69.4	7.8	21.1

**Table 6:** DNA methylation scores by Methylation Rank.

DNA Methylation Rank	Number of Regions (*n*)	Methylation Score (Mean ± SD)
Hyper-methylation	125	0.93 ± 0.04
Hypo-methylation	38	0.20 ± 0.07
Mild methylation	14	0.05 ± 0.02
No methylation	3	0.00 ± 0.00

**Table 7:** Logistic regression analysis of factors associated with (rs5030619) mutation in the *MED12* gene.

Variable	Odds Ratio (OR)	95% CI	*p*-Value
Age (years)	1.04	1.01–1.07	0.012
BMI (kg/m^2^)	1.09	1.03–1.16	0.004
Multiple fibroids	2.31	1.28–4.17	0.005
Intramural location	1.88	1.02–3.44	0.041
Family history	2.74	1.36–5.52	0.003

**Table 8:** Linear regression analysis of DNA methylation levels in cases.

Predictor	β Coefficient	Standard Error	*p*-Value
*MED12* mutation	+4.6	1.2	<0.001
Age	+0.08	0.03	0.011
BMI	+0.15	0.05	0.006

## Data Availability

All data generated or analyzed during this study are included in this published article.

## Abbreviations

The following abbreviations are used in this manuscript:

*MED12*	Mediator Complex Subunit 12
BMI	Body Mass Index
DNA	Deoxy Ribonucleic Acid
PCR	Polymerase Chain Reaction
ELISA	Enzyme-Linked Immunosorbent Assay
FSH	Follicle-Stimulating Hormone
